# Supplementary material for: Abnormal mGluR-mediated synaptic plasticity and autism-like behaviours in Gprasp2 mutant mice
Source: Nat Commun. 2019 Mar 29;10:1431. doi: 10.1038/s41467-019-09382-9 (PMC6440958; doi:10.1038/s41467-019-09382-9)

Supplementary Information

Abnormal mGluR-mediated synaptic plasticity and autism-like behaviours in *Gprasp2* mutant mice

Edfawy M et al.

Supplementary Table 1 – GPRASP gene family expression in the human brain

Gene	Transcript distribution#	Relative expression level in main tissue*
GPRASP1	Wide expression in CNS; ovary; pituitary	+++++
GPRASP2	**Wide expression in CNS; ovary; pituitary**	**+++**
GPRASP3	Wide expression in CNS; ovary; pituitary	++
GPRASP4	Ovary; pituitary; prostate	++
GPRASP5	Wide expression in most tissues. Lower in CNS	+
GPRASP6	Wide expression in most tissues. Lower in CNS	++++
GPRASP7	Wide expression in most tissues. Lower in CNS	++++
GPRASP8	Wide expression in most tissues. Lower in CNS	++
GPRASP9	Pituitary, ovary; thyroid	++++
GPRASP10	Wide expression in most tissues. Lower in CNS	++

# - If several organs, only top 3 are indicated
* - Summary data taken from to the Genotype-Tissue Expression (GTEx) project - The Genotype-Tissue Expression (GTEx) Project was supported by the Common Fund of the Office of the Director of the National Institutes of Health, and by NCI, NHGRI, NHLBI, NIDA, NIMH, and NINDS.

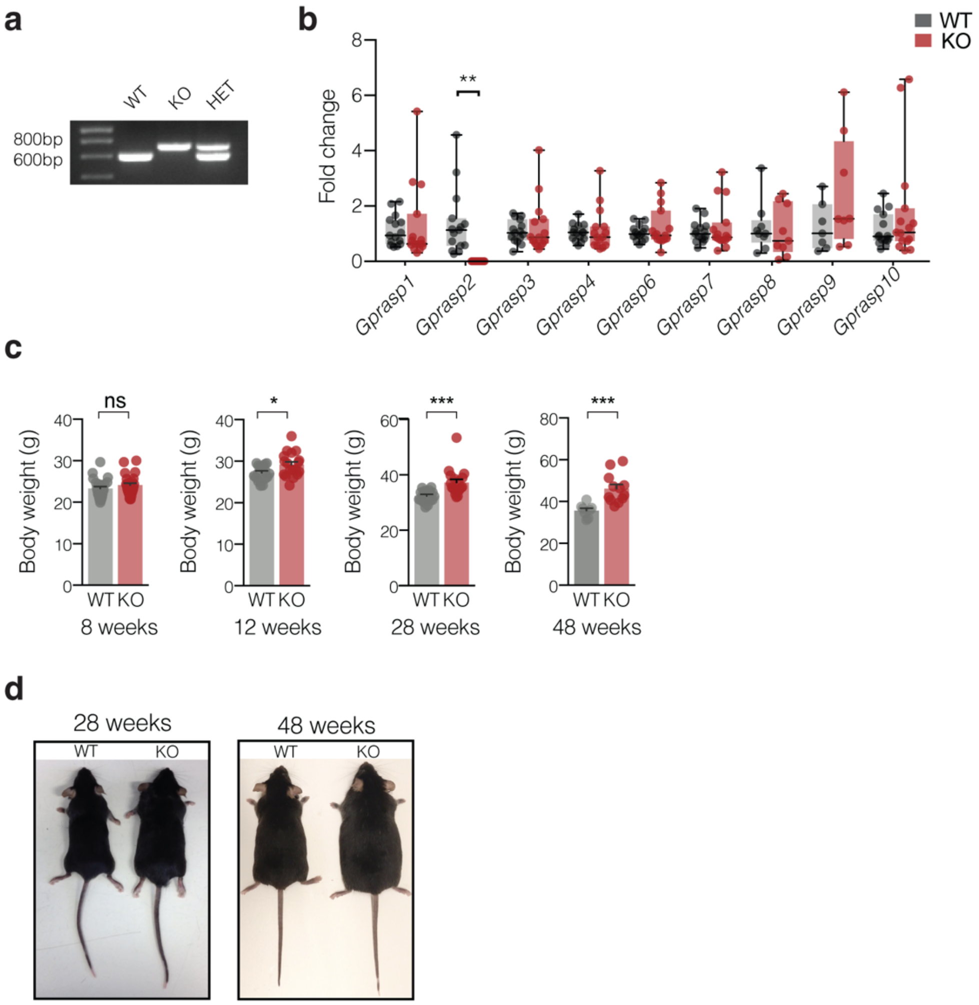

Supplementary Figure 1 | Increased body weight in adult *Gprasp2* KO mice. a, PCR genotyping of *Gprasp2*$^{-/y}$, *Gprasp2*$^{+/y}$ and a *Gprasp2*$^{+/-}$ heterozygous female. **b**, *Gprasp* family members are not significantly altered in the brain of Gprasp2$^{-/y}$ mice; Gprasp5 failed to amplify; n = 7-15; one-way ANOVA test with Sidak *post hoc* test **c**, Increased body weight in Gprasp2 KO started approximately following 12-weeks of age and increased during adulthood; WT n = 8-22, KO n = 12-24; two-tailed t-test. **d**, Representative images from 28- and 48-week old WT and Gprasp2 KO mice. Data is presented as means ± s.e.m. Statistical significance: *p<0.05, ***p<0.001.

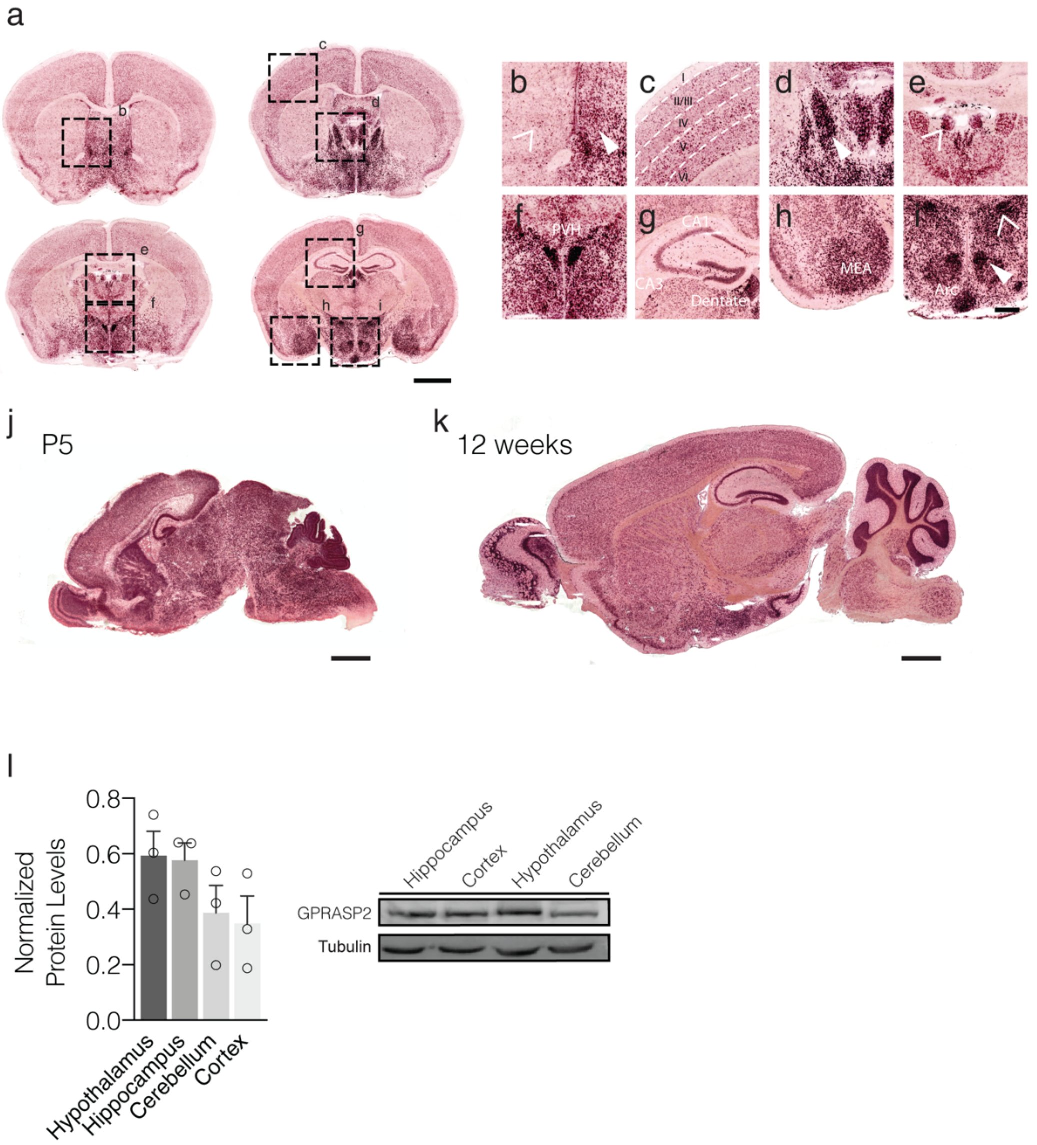

Supplementary Figure 2 | *In situ* mRNA labelling of GPRASP2 in the mouse brain. a, Coronal sections of P15 mice hybridized with GPRASP2 antisense probe. **b**, Expression of GPRASP2 mRNA in the medial dial septum, with the solid arrowhead pointing to septal region and the open arrowhead pointing to medial striatum. **c**, GPRASP2 transcript is sparsely detected in the cortex. **d**, GPRASP2 transcript is strongly detected in thalamic nuclei. **e**, Arrowhead pointing to GPRASP2 mRNA labelling in the medial habenula. **f**, Strong expression of GPRASP2 mRNA in the paraventricular nucleus of the hypothalamus (PVH). **g**, Strong expression of GPRASP2 transcripts in CA1, CA3 and dentate gyrus of the hippocampus. **h**, Strong labelling is detected in medial amygdala nucleus (MEA). **i**, GPRASP2 transcript is highly expressed in the dorsal medial hypothalamic nucleus (open arrow), the ventromedial hypothalamus (arrowhead) and in the arcuate nucleus of the hypothalamus (Arc). High magnification images of the squared region in the left panels (Scale bar, 2 mm) are shown in the adjacent right panels (Scale bar, 500 µm). **j**-**k**, Dig-labelled *in situ* hybridization using parasagittal mouse brain sections showing GPRASP2 mRNA expression in P5 (**j**) and 12-week-old mouse (**k**) Scale bar, 1 mm. **l**, Expression levels of Gprasp2 is highest in the hypothalamus and hippocampus of the adult mouse brain, lower levels are seen in the cortex and cerebellum; n = 3.

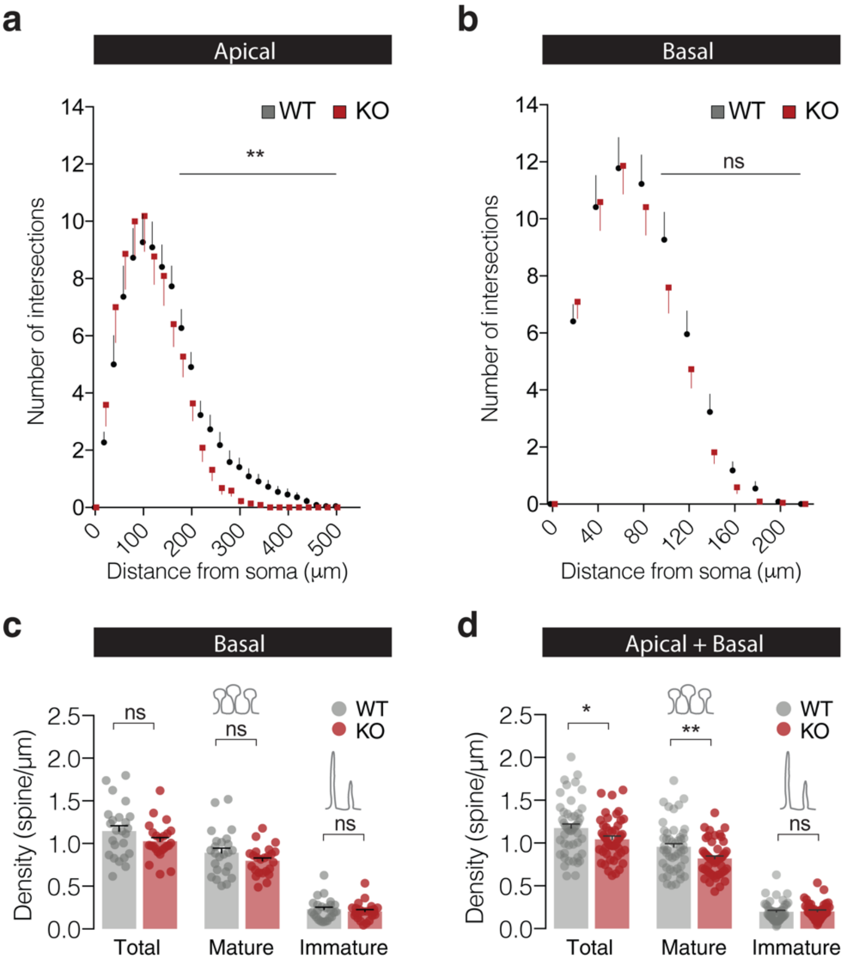

Supplementary Figure 3 | Structural alterations in *Gprasp2* knockout mice are most pronounced in apical dendrites. a-b, Sholl analysis reveals decreased neuronal complexity in the apical (**a**) but not basal (**b**) arborization of *Gprasp2*$^{-/y}$ CA1 pyramidal neurons; WT n=22/3 neurons/mice, KO n=22/3 neurons/mice; two-way repeated measures ANOVA. **c-d**, Basal dendrites display trend towards lower spine density (**c**); WT n=24/3 branches/mice, KO n=24/3 branches/mice. Combining data from apical dendrites (from Figure 3b) with spine density from basal dendrites reveals a reduction in total and mature spine density (**d**); WT n=48/3 branches/mice, KO n=48/3 branches/mice two-tailed *t*-test. All data is presented as mean ± s.e.m. Statistical significance: *p<0.05, **p<0.01.

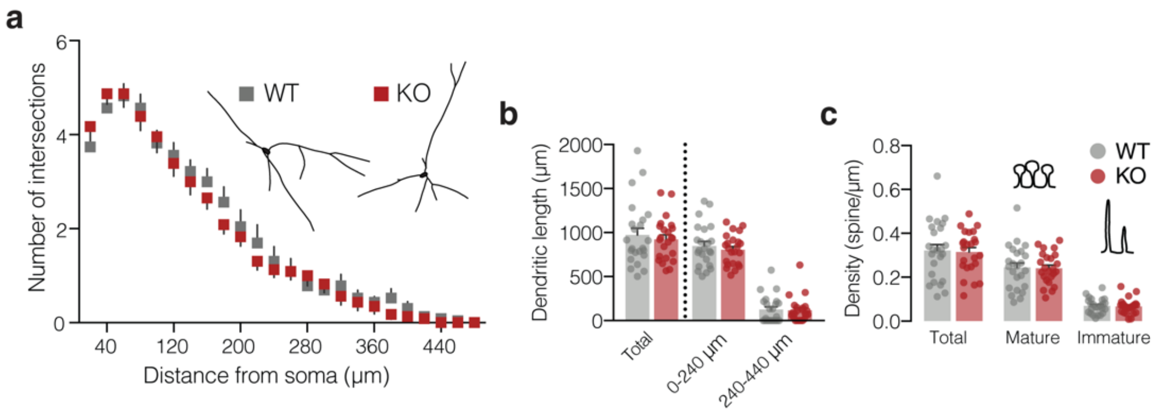

Supplementary Figure 4 | No significant differences in hypothalamic neuron morphology in Gprasp2$^{-/y}$ mice. a-b, Sholl analysis reveals mostly intact neuronal complexity (**a**) and dendritic length (**b**) in hypothalamic neurons from *Gprasp2$^{-/y}$* mice; WT n=23/3 neurons/mice, KO n=23/3 neurons/mice; two-way repeated measures ANOVA. **c**, Similar spine density in hypothalamic neurons from *Gprasp2$^{-/y}$* and WT littermate controls; WT n=23/3 branches/mice, KO n=23/3 branches/mice; two-tailed t-test. All data is presented as mean ± s.e.m. Statistical significance: *p<0.05, **p<0.01.

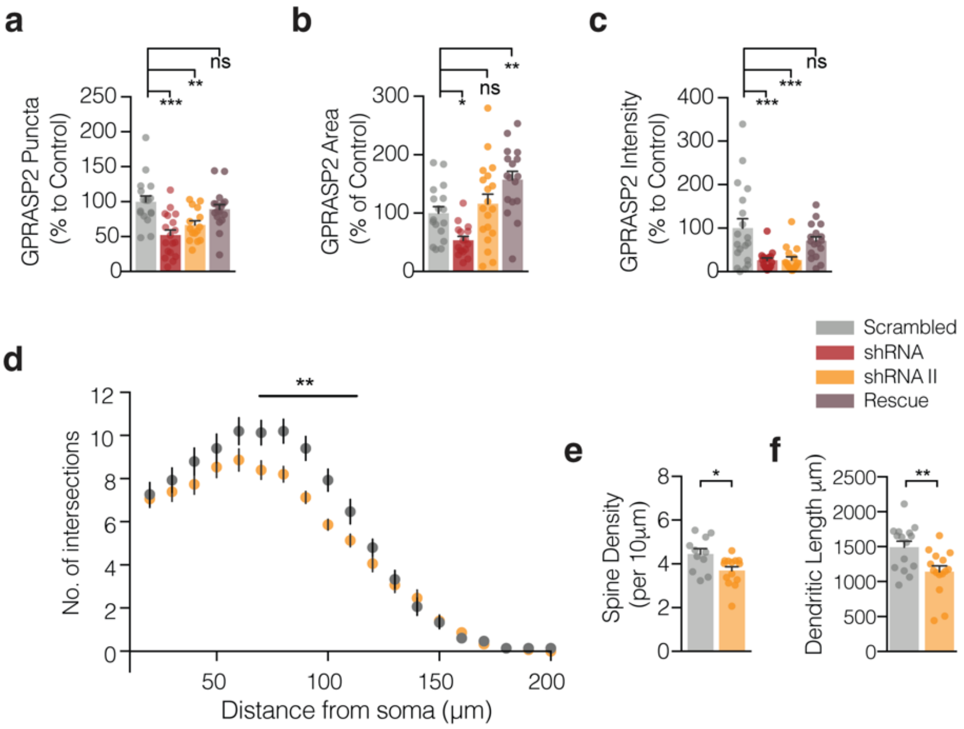

Supplementary Figure 5 | shRNA reduction of GPRASP2. a-c, Cultured hippocampal neurons were transfected with scramble shRNA (Scramble), shRNA (most effective shRNA), shRNA II (a less effective shRNA) and rescue experiment (GFP-GPRASP2 + shRNA) and the levels of GPRASP2 puncta number (**a**), size (**b**) and intensity (**c**) were analysed; scramble n = 17, shRNA n = 18, shRNA II n = 15, rescue n=17, from three independent preparations; one-way ANOVA test followed by Dunnett multiple comparisons test. **d-f,** A less effective shRNA II, targeting a different region of GPRASP2 was determined to also significantly decrease neuronal complexity(**d**), spine density (**e**) and total dendritic length (**f**) in cultured hippocampal neurons; neuronal morphology WT n = 15, KO n= 15, repeated measures two-way ANOVA effect of treatment **p<0.01; Spine density WT n = 11, KO n = 14, unpaired two-tailed t-test. Data is presented as means ± s.e.m. Statistical significance: *p<0.05, **p<0.01 and ***p<0.001.

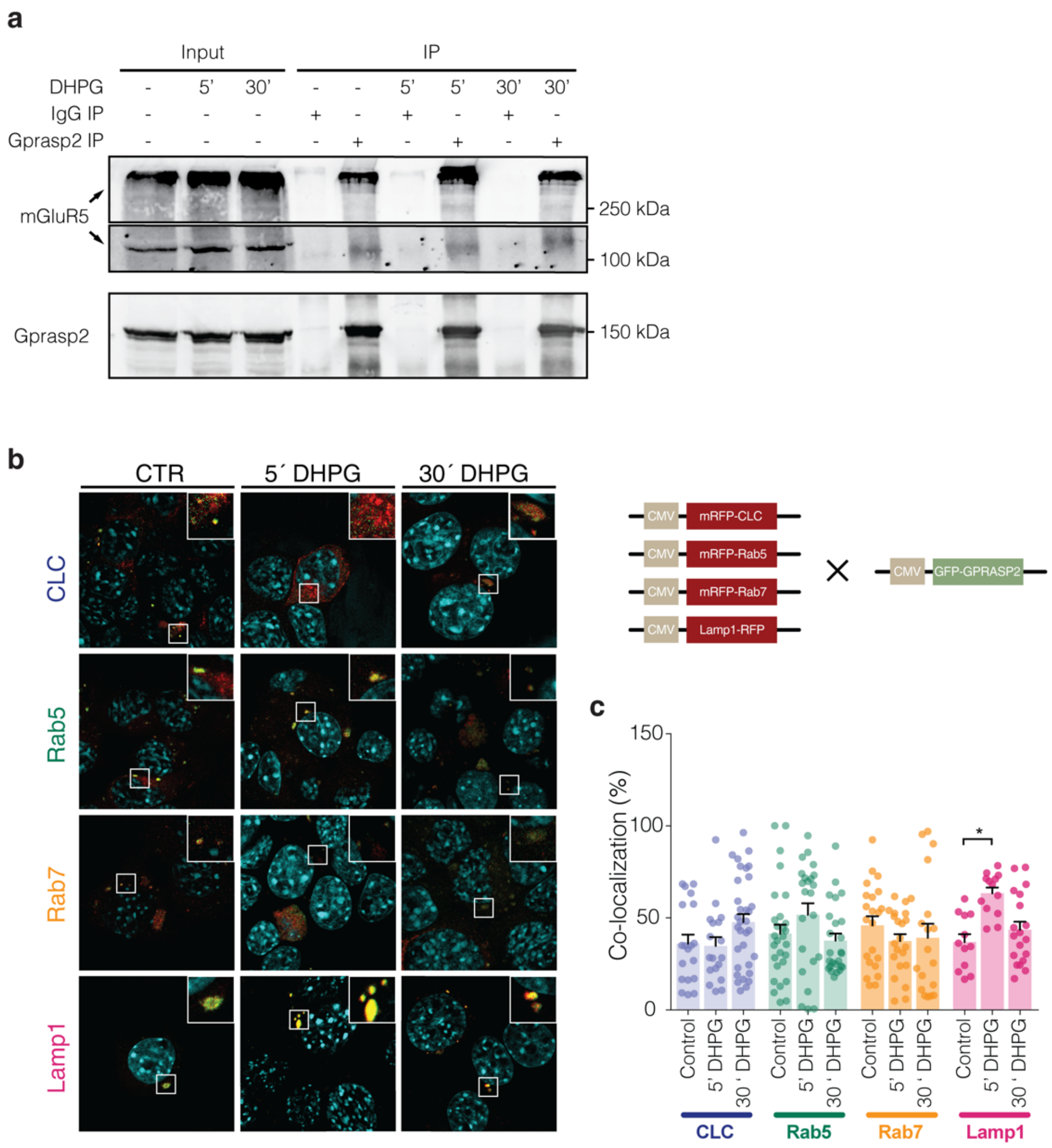

Supplementary Figure 6 | GPRASP2-mGluR$_5$ interaction and localization with endocytic machinery. a, Representative experiment showing mGluR$_5$ co-immunoprecipitation following GPRASP2-GFP pulldown in transfected HT-22 cells under control conditions, 5-minute and 30-minute DHPG stimulation. **b**, Co-localization of GPRASP2-GFP (green) was analysed as overlay (yellow) with clathrin light chain (mRFP-CLC), Rab5 (mRFP-Rab5), Rab7 (mRFP-Rab7) or Lamp1 (Lamp1-RFP) (red channels) in, control conditions after 5- or after 30-minute stimulation with DHPG. **c**, Quantification of percent co-localization shows increased GPRASP2 overlap with Lamp1 following 5´ DHPG stimulation; n = 12–33 transfected cells per condition; one-way ANOVA with Bonferroni post-hoc comparison; scale bar 10 μm. Data is presented as means ± s.e.m. Statistical significance: *$p<0.05$.

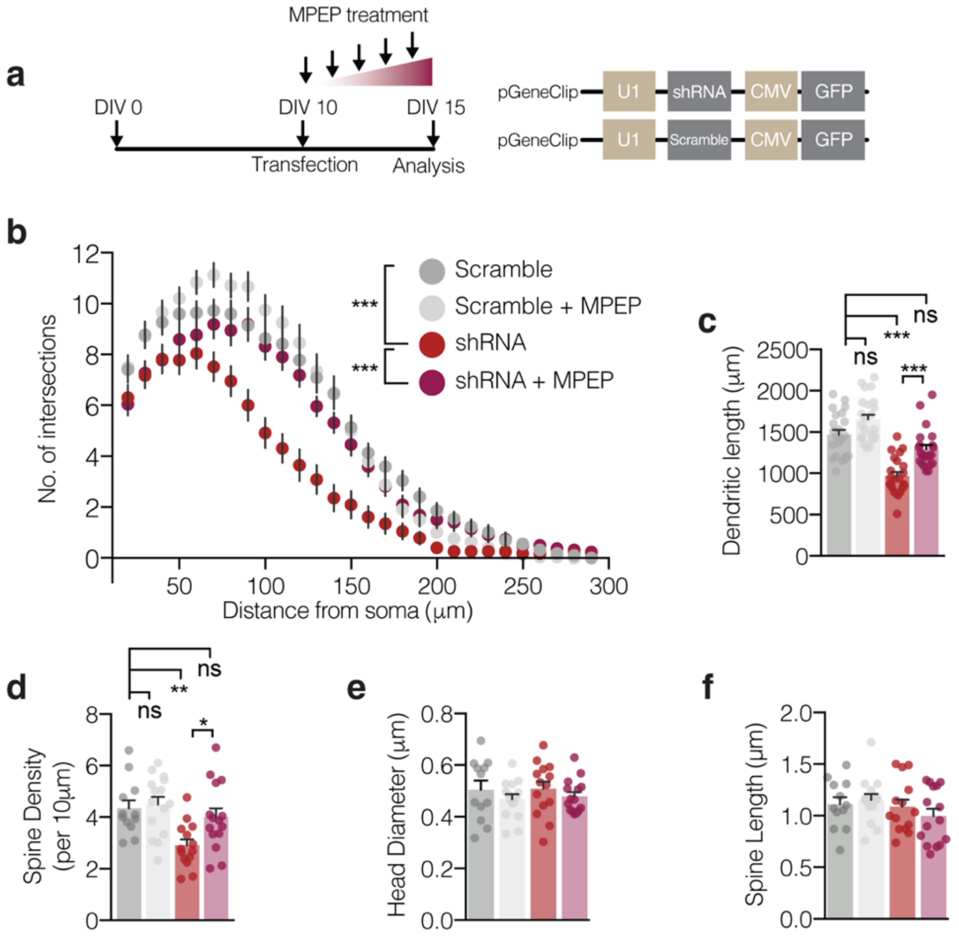

Supplementary Figure 7 | MPEP blocks *Gprasp2* knockdown-mediated effects on neuronal morphology. a, Cultured hippocampal neurons were transfected with scramble shRNA (Scramble) or shRNA against *Gprasp2* (shRNA) at DIV10. From DIV10 through DIV14, MPEP (20 µM) was added to the culture media and neurons were analysed at DIV15. **b**, Sholl analysis reveals reduced dendritic complexity in shRNA-transfected neurons when compared to Scramble (effect of treatment ***p<0.001), MPEP treatment significantly increased neuronal complexity in shRNA transfected neurons (effect of treatment ***p<0.01); n=22-23 neurons per condition, from three independent preparations; two-way repeated measures ANOVA with Bonferroni *post hoc* test. **c**, GPRASP2 knockdown-mediated decrease in dendritic length is rescued by MPEP treatment; n=22-23 neurons per condition, from three independent preparations; one-way ANOVA test followed by Tukey multiple comparison test. **d-f,** GPRASP2 knockdown-mediated decrease in spine density (**d**) is rescued by MPEP treatment, but not changes were observed in either spine head diameter (**e**) or spine length (**f**); n=12-15 dendritic segments sampled from neurons in three independent preparations; one-way ANOVA test followed by Tukey multiple comparison test. Data is presented as means ± s.e.m. Statistical significance: *p<0.05, **p<0.01 and ***p<0.001.

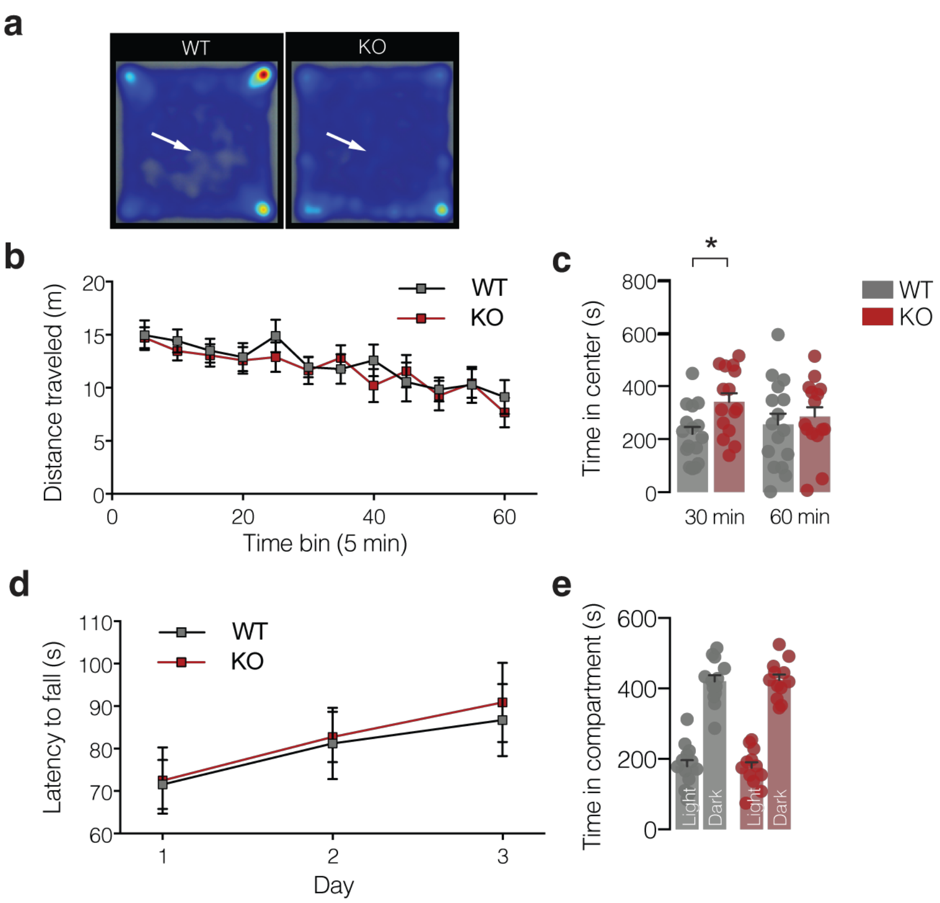

Supplementary Figure 8 | *Gprasp2$^{-/y}$* mice display normal motor and anxiety-like behaviour. a-c, Representative heat maps (**a**) in the open field test; spontaneous locomotor activity (**b**) is not significantly different between *Gprasp2$^{-/y}$* mice and WT littermate controls, time in center of the field (**c**) is increased in the first 30-minutes of the open field test; WT n = 16, KO n = 15. **d**, *Gprasp2$^{-/y}$* mice displayed normal motor learning behaviour in rotarod test; WT n = 10, KO n = 10. **e**, No significant difference between genotypes in dark-light box emergence test; WT n = 13; KO n = 12. Two-way repeated measures ANOVA with Bonferroni *post hoc* test for (**a** and **d**), one-way ANOVA with Bonferroni *post hoc* for (**c** and **e**). Data is presented as means ± s.e.m. Statistical significance: $^{*}p<0.05$.

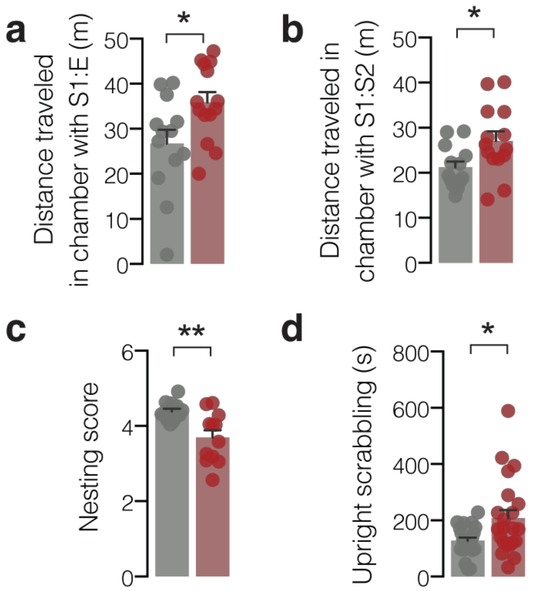

Supplementary Figure 9 | Social-induced hyperactivity, perturbed nesting behaviour and stereotypical activity. a-b, *Gprasp2*$^{-/y}$ mice display increased distance travelled in the three-chamber social behaviour test when compared to controls; WT n = 13; KO, n = 14. **c**, Impaired nest building in *Gprasp2*$^{-/y}$ mice; WT n = 13, KO n = 12. **d**, *Gprasp2*$^{-/y}$ mice engage in more upright scrabbling events during an open field session; WT n = 16, KO n = 15. Two-tailed t-test; data is presented as means ± s.e.m. Statistical significance: *p<0.05, **p<0.01.

Supplementary Figure 10

Fig 1c

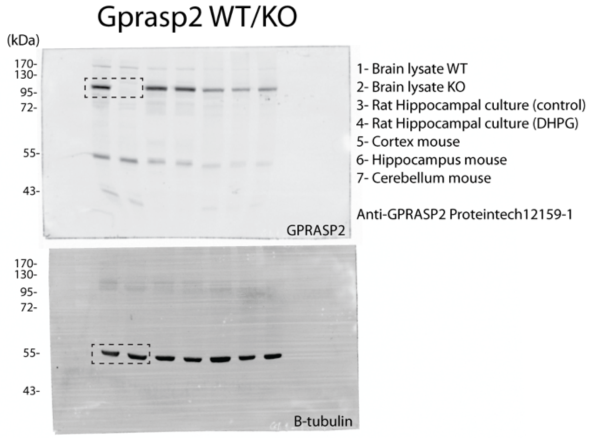

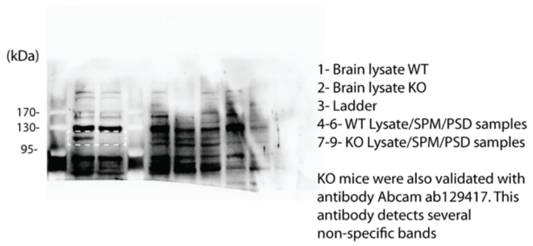

Fig 1d

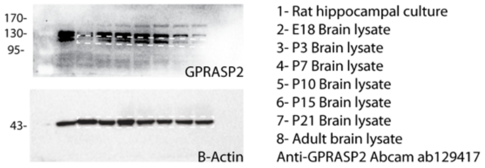

Fig 2j

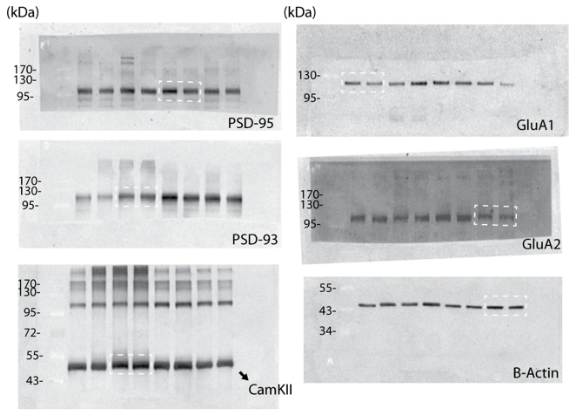

Sup. Fig 2l

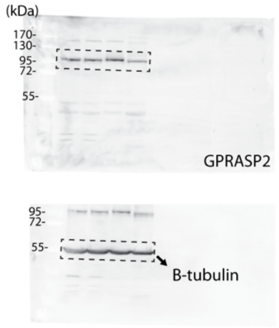

Sup. Fig 6a

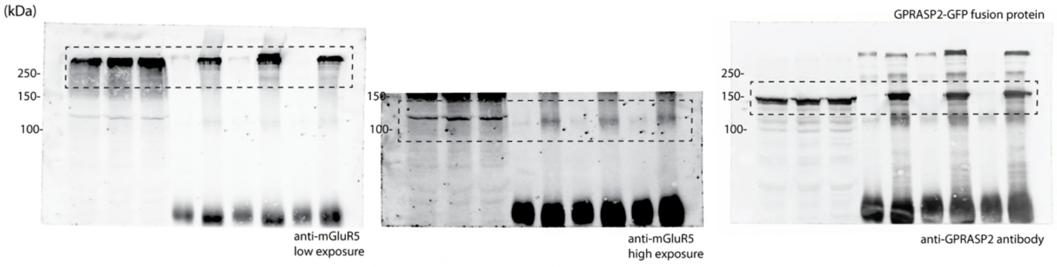

Supplement: Supplementary file 1 — Supplementary Information [file 41467_2019_9382_MOESM1_ESM.pdf]
