# Supplementary material for: Abnormal mGluR-mediated synaptic plasticity and autism-like behaviours in Gprasp2 mutant mice
Source: Nat Commun. 2019 Mar 29;10:1431. doi: 10.1038/s41467-019-09382-9 (PMC6440958; doi:10.1038/s41467-019-09382-9)
Supplement: Supplementary file 3 — Reporting Summary [file 41467_2019_9382_MOESM3_ESM.pdf]

nature research

Corresponding author(s): João Peça

Last updated by author(s): Feb 28, 2019

Reporting Summary

Nature Research wishes to improve the reproducibility of the work that we publish. This form provides structure for consistency and transparency in reporting. For further information on Nature Research policies, see Authors & Referees and the Editorial Policy Checklist.

Statistics

For all statistical analyses, confirm that the following items are present in the figure legend, table legend, main text, or Methods section.

n/a	Confirmed	
☐	☒	The exact sample size (*n*) for each experimental group/condition, given as a discrete number and unit of measurement
☐	☒	A statement on whether measurements were taken from distinct samples or whether the same sample was measured repeatedly
☐	☒	The statistical test(s) used AND whether they are one- or two-sided<br>*Only common tests should be described solely by name; describe more complex techniques in the Methods section.*
☒	☐	A description of all covariates tested
☐	☒	A description of any assumptions or corrections, such as tests of normality and adjustment for multiple comparisons
☐	☒	A full description of the statistical parameters including central tendency (e.g. means) or other basic estimates (e.g. regression coefficient) AND variation (e.g. standard deviation) or associated estimates of uncertainty (e.g. confidence intervals)
☐	☒	For null hypothesis testing, the test statistic (e.g. *F*, *t*, *r*) with confidence intervals, effect sizes, degrees of freedom and *P* value noted<br>*Give P values as exact values whenever suitable.*
☒	☐	For Bayesian analysis, information on the choice of priors and Markov chain Monte Carlo settings
☒	☐	For hierarchical and complex designs, identification of the appropriate level for tests and full reporting of outcomes
☒	☐	Estimates of effect sizes (e.g. Cohen's *d*, Pearson's *r*), indicating how they were calculated

Our web collection on statistics for biologists contains articles on many of the points above.

Software and code

Policy information about availability of computer code

Data collection	Confocal images: ZEN 2012, Ephys data: pClamp 10,7, Behavioral data: Ethovision XT and The Observer XT 9, Neuronal Morphology: Neurolucida v10.42
Data analysis	GraphPad Prism v7, Matlab 2017b, Neurolucida Neuroexplorer v10.42, FIJI/ImageJ, Excel v16, Clampfit v10.7

For manuscripts utilizing custom algorithms or software that are central to the research but not yet described in published literature, software must be made available to editors/reviewers. We strongly encourage code deposition in a community repository (e.g. GitHub). See the Nature Research guidelines for submitting code & software for further information.

Data

Policy information about availability of data

All manuscripts must include a data availability statement. This statement should provide the following information, where applicable:

- Accession codes, unique identifiers, or web links for publicly available datasets
- A list of figures that have associated raw data
- A description of any restrictions on data availability

The data, computer scripts, protocols and biological materials included in this study are available from the corresponding author upon reasonable request.

Field-specific reporting

Please select the one below that is the best fit for your research. If you are not sure, read the appropriate sections before making your selection.

☒ Life sciences ☐ Behavioural & social sciences ☐ Ecological, evolutionary & environmental sciences

Life sciences study design

All studies must disclose on these points even when the disclosure is negative.

Sample size	Sample sizes were selected based on data from similar studies present in the literature.
Data exclusions	No data was excluded from the analysis.
Replication	All the experimental findings were reliably reproduced and the number of replicates are indicated in the corresponding figure legends.
Randomization	Randomization was performed and the number of animals per group in indicated in the figure legends.
Blinding	Experimenters were blinded to the genotype of the animals.

Reporting for specific materials, systems and methods

We require information from authors about some types of materials, experimental systems and methods used in many studies. Here, indicate whether each material, system or method listed is relevant to your study. If you are not sure if a list item applies to your research, read the appropriate section before selecting a response.

Materials & experimental systems

n/a	Involved in the study	
☐	☒	Antibodies
☐	☒	Eukaryotic cell lines
☒	☐	Palaeontology
☐	☒	Animals and other organisms
☒	☐	Human research participants
☒	☐	Clinical data

Methods

n/a	Involved in the study	
☒	☐	ChIP-seq
☒	☐	Flow cytometry
☒	☐	MRI-based neuroimaging

Antibodies

Antibodies used

Primary antibodies: anti-PSD-95 (6G6-1C9, lot#QA210648, clone 6G6-1C9, 1:1000; ThermoFisher Scientific)

anti-PSD-93 (N18/275-284, lot#75-284, clone N18/28,1:1000 NeuroMab)

anti-CAMK2a (6G9, lot# T286/287, clone 6G9, 1:1000, Sigma-Aldrich)

anti-GluA1 (AB1504, lot# 2965121, 1:1000, Millipore)

anti-GluA2 (MAB397, lot#3070189, clone 6c4, 1:1000, Millipore)

anti-GPRASP2 (ab129417, lot#GR88574-5, 1:100; Abcam

anti-GPRASP2 (12159-1-AP, lot#1, 1:1000; Proteintech)

Anti-mGluR5 intracellular N-terminus antibody (AGC-007, lot# AGC007AN0725, 1:100, Alomone labs)

anti-[illegible]-tubulin (T5168,lot# 072M4809, clone B-5-1-2, 1:20,000; Sigma-Aldrich)

anti-β-actin (A5441, lot# 030M4788, clone AC-15,1:5000; Sigma-Aldrich)

anti-MAP2 (ab5392, lot# 6R3242762-3,1:5000, Abcam)

anti-mGluR1/5 (75-116, lot# 443.2KS.76,1:200, NeuroMab),

anti-VGLUT1 (AB5905, lot# 2517839, 1:5000, Millipore).

Secondary antibodies: Peroxidase-conjugated Affinipure Donkey Anti-mouse (715-035-150, lot# 127332,1:10000, Jackson ImmunoResearch

Peroxidase-conjugated Affinipure Donkey Anti-rabbit (711-035-152, lot# 121596,1:10000, Jackson ImmunoResearch

Alexa 568-conjugated anti-mouse (A11004, lot# 1862187,1:500, ThermoFisher Scientific)

	Alexa 488-conjugated anti-rabbit (A11008, lot# 1981125, 1:500, ThermoFisher Scientific)<br><br>AMCA-conjugated anti-chicken (103-155-155, lot#136324, 1:200, Jackson ImmunoResearch)<br><br>Alexa 647-conjugated anti-guinea pig (A21450,lot# 1841758, 1:500 , ThermoFisher Scientific)
Validation	All antibodies used were commercial and validated in previous studies (see manufacturer's website and article references).<br><br>https://www.thermofisher.com/antibody/product/PSD-95-Antibody-clone-6G6-1C9-Monoclonal/MA1-045<br>http://neuromab.ucdavis.edu/datasheet/N18_28.pdf<br>https://www.sigmaaldrich.com/catalog/product/sigma/c265?lang=pt&region=PT<br>http://www.merckmillipore.com/PT/en/product/Anti-Glutamate-receptor-1-Antibody,MM_NF-AB1504?ReferrerURL=https%3A%2F%2Fwww.google.com%2F<br>http://www.merckmillipore.com/PT/en/product/Anti-Glutamate-Receptor-2-Antibody-extracellular-clone-6C4,MM_NF-MAB397?ReferrerURL=https%3A%2F%2Fwww.google.com%2F&bd=1<br>https://www.abcam.com/gprasp2-antibody-ab129417.html<br>https://www.ptglab.com/products/GPRASP2-Antibody-12159-1-AP.htm<br>https://www.alomone.com/p/anti-mglur5-extracellular/AGC-007<br>https://www.sigmaaldrich.com/catalog/product/sigma/t5168?lang=pt&region=PT<br>https://www.sigmaaldrich.com/catalog/product/sigma/a5441?lang=pt&region=PT<br>https://www.abcam.com/map2-antibody-ab5392.html<br>https://www.antibodiesinc.com/products/mglur1-5-group-i-glutamate-receptor-n75-33<br>http://www.merckmillipore.com/PT/en/product/Anti-Vesicular-Glutamate-Transporter-1-Antibody,MM_NF-AB5905?ReferrerURL=https%3A%2F%2Fwww.google.com%2F<br><br>Secondary antibodies:<br><br>https://www.jacksonimmuno.com/catalog/products/715-035-150<br>https://www.jacksonimmuno.com/catalog/products/711-035-152<br>https://www.thermofisher.com/antibody/product/Goat-anti-Mouse-IgG-H-L-Cross-Adsorbed-Secondary-Antibody-Polyclonal/A-11004<br>https://www.thermofisher.com/antibody/product/Goat-anti-Rabbit-IgG-H-L-Cross-Adsorbed-Secondary-Antibody-Polyclonal/A-11008<br>https://www.jacksonimmuno.com/catalog/products/103-155-155<br>https://www.thermofisher.com/antibody/product/Goat-anti-Guinea-Pig-IgG-H-L-Highly-Cross-Adsorbed-Secondary-Antibody-Polyclonal/A-21450

Eukaryotic cell lines

Policy information about cell lines

Cell line source(s)	HT-22 cells were a kind gift, from Prof. Ernst Wagner lab, Ludwig-Maximillian University, Munich, Germany.<br>R01 Embryonic cell line were a kind gift from Prof. Andras Nagy, The Lunenfeld-Tanenbaum Research Institute, Toronto, Canada.
Authentication	None of the cell lines used were authenticated.
Mycoplasma contamination	All cell lines employed were regularly tested for mycoplasm contamination and no contamination was found troughout this study.
Commonly misidentified lines<br>(See ICLAC register)	n.a.

Animals and other organisms

Policy information about studies involving animals; ARRIVE guidelines recommended for reporting animal research

Laboratory animals	We used mus musculus of the C57/BL6 strain, transgenic mice expressing B-Actin:Cre and genetically engineered GPRASP2 mice. The age, sex and gender of all animals used is indicated in the figure legends and methods section.
Wild animals	n.a.
Field-collected samples	n.a.
Ethics oversight	ORBEA - Institutional Animal Welfare Body of the University of Coimbra/CNC: Project reference number 127/2016).<br>DGAV - Portuguese Regulatory Agency: Project reference number 0421/2016).

Note that full information on the approval of the study protocol must also be provided in the manuscript.
